# Semi-Theoretical Modeling and Experimental Validation of the Extrusion Swell Ratio of Highly Concentrated Silver Paste in Micro-Extrusion

**DOI:** 10.3390/mi17070855

**Published:** 2026-07-17

**Authors:** Zhijie Huang, Shixiong Wu, Zhichao Yuan, Zeyu Wang, Cuimin Sun, Hui You

**Affiliations:** 1 School of Mechanical Engineering, Guangxi University, Nanning 530004, China; 2511401002@st.gxu.edu.cn (Z.H.); 2511300079@st.gxu.edu.cn (Z.Y.); 2511390203@st.gxu.edu.cn (Z.W.); 2 School of Chemistry and Chemical Engineering, Shanghai Jiao Tong University, Shanghai 200240, China; 3 School of Computer and Electronic Information Engineering, Guangxi University, Nanning 530004, China; cmsun@gxu.edu.cn; 4 Key Laboratory of Future Intelligent Manufacturing Technologies for High-end Equipment, Ministry of Education, Fuyao University of Science and Technology, Fuzhou 350109, China

**Keywords:** conductive silver paste, micro-extrusion printing, direct ink writing, extrusion swell, Herschel–Bulkley model, nozzle length, free-filament size estimation

## Abstract

In micro-extrusion and direct ink writing, the nozzle outlet diameter is often used to estimate the deposited line width or free-filament diameter. However, highly loaded conductive silver pastes may exhibit pronounced extrusion swell after leaving the nozzle, resulting in a filament diameter larger than the nozzle inner diameter. To quantify this deviation, this study proposes a single-parameter semi-theoretical correction model based on radial force balance at the nozzle exit, integrating Herschel–Bulkley yield stress–shear-thinning rheology with a finite-deformation description. The exit radial stress is derived from pressure-driven circular tube flow, while the post-exit radial expansion is balanced against atmospheric constraint. A comprehensive correction force constant, C, is introduced to account for wall-induced energy dissipation, particle-structure rearrangement, residual elastic recovery, and model simplifications. After calibration using a transition-swelling nozzle, C was determined as 1.03 × 10^−2^ N. The model was applied to six nozzle diameters and four nozzle length–pressure conditions. For Nozzles 1–4 with significant swelling, the mean absolute percentage error was 5.31%, while the overall error for all six nozzles was 11.84%, mainly due to overestimation for the nearly non-swelling Nozzle 6. For varying nozzle lengths, the error was 5.20%, and both experimental and predicted swell ratios decreased with increasing effective nozzle length. The model provides a semi-theoretical tool for estimating free-filament dimensions and analyzing nozzle-length effects, primarily under pronounced-swell conditions. Its predictive capability becomes limited as the swell ratio approaches unity, where additional corrections for wall slip, relaxation, and the zero-swell boundary are required.

## 1. Introduction

Direct ink writing (DIW) and micro-extrusion printing are important material-extrusion-based additive manufacturing techniques. In these processes, pastes, gels, or inks with certain viscoelastic or yield-stress characteristics are continuously extruded through fine nozzles under pneumatic, piston-driven, or screw-driven actuation and are deposited along predefined paths to form two-dimensional patterns or three-dimensional structures. Compared with droplet-based deposition methods such as inkjet printing, DIW and micro-extrusion printing can process material systems with higher viscosity and higher solid loading. Therefore, they have been widely applied to the fabrication of ceramics, metals, polymer composites, conductive inks, bioelectronic devices, and flexible functional structures [1,2,3,4,5,6]. During printing, the rheological properties of the ink, nozzle geometry, and processing parameters jointly determine whether the material can be extruded stably, maintain its shape after deposition, and achieve the required dimensional accuracy [4,5,6].

Conductive silver paste is a widely used conductive material system in printed electronics, flexible interconnects, wearable sensors, and miniaturized functional devices. Previous studies have shown that conductive inks based on silver nanoparticles or silver flakes can be processed by direct writing to fabricate flexible, stretchable, or three-dimensional conductive microstructures, showing considerable potential in soft electronics, antennas, sensors, and bioelectronic devices [7,8,9,10,11,12]. For highly loaded conductive silver pastes, a high silver-particle content helps reduce the electrical resistance of sintered conductive traces, improve the continuity of conductive pathways, and decrease the process complexity associated with repeated printing. However, such pastes are typically composed of a high volume fraction of particles, a resin or binder phase, solvents, and additives, and thus belong to the category of concentrated suspension-based complex fluids. Their flow behavior cannot be adequately described by the simple Newtonian-fluid assumption. Instead, they commonly exhibit yield stress, shear thinning, thixotropic recovery, wall slip, and certain viscoelastic responses [13,14,15,16,17,18,19,20,21]. Consequently, the printed line width or the diameter of a freely extruded silver-paste filament cannot be determined simply from the nozzle outlet diameter. Rather, it depends on the rheological parameters of the material, the confined flow state inside the nozzle, and the deformation recovery behavior after exiting the nozzle.

In practical micro-extrusion processes, the diameter of the freely extruded filament is often larger than the inner diameter of the nozzle after the paste leaves the nozzle. This phenomenon is commonly referred to as extrusion swell or die swell. For the fabrication of fine conductive traces, extrusion swell directly causes line-width deviation, reduces pattern dimensional accuracy, and increases the uncertainty in nozzle selection and process-parameter optimization. Classical theories of die swell are mainly derived from studies of polymer melts and viscoelastic fluids. These theories generally attribute transverse swelling of the extrudate to the release of recoverable deformation, first normal stress difference, or elastic energy accumulated during confined flow inside the channel [22,23]. For yield-stress and shear-thinning fluids such as Herschel–Bulkley fluids, previous studies have analyzed pressure loss, yield-region distribution, and free-surface evolution in extrusion channels mainly through numerical simulations [24,25]. These studies provide an important basis for understanding outlet swelling in non-Newtonian fluids. However, highly loaded conductive silver pastes differ substantially from homogeneous polymer melts or idealized yield-stress fluids. Their internal particle networks, binder-phase recovery, wall-induced energy dissipation, and particle rearrangement jointly influence the outlet swelling process, making it difficult to directly apply conventional die-swell models to the prediction of free-filament dimensions in silver-paste micro-extrusion.

Existing approaches for predicting extrudate dimensions or extrusion swell mainly include empirical correlations, classical die-swell theories, and numerical free-surface simulations. Empirical correlations commonly relate the deposited line width or filament diameter to variables such as nozzle diameter, feeding pressure, printing speed, and nozzle height [5,6,13]. Although convenient, their fitted parameters are generally dependent on the material formulation, equipment, and operating conditions, and they provide a limited description of the deformation release at the nozzle exit. Classical die-swell theories, developed primarily for homogeneous polymer melts and viscoelastic fluids, attribute swelling to recoverable deformation, normal-stress differences, or stored elastic energy [22,23]. However, their direct application to highly concentrated silver pastes is difficult because these materials exhibit yield stress, shear thinning, particle-network rearrangement, wall slip, and thixotropic recovery. Numerical free-surface simulations can provide detailed flow and stress fields [24,25], but usually require complex constitutive equations, boundary conditions, and considerable computational effort. Therefore, a concise model that incorporates the principal rheological, geometric, and processing variables with limited experimental calibration is still needed.

To address this gap, this study develops a single-parameter semi-theoretical model for the free-filament swelling of highly concentrated conductive silver paste during micro-extrusion. The model combines the Herschel–Bulkley constitutive relation, pressure-driven flow analysis, and radial force balance at the nozzle exit. The feeding pressure, nozzle radius, effective nozzle length, and independently measured rheological parameters are explicitly incorporated, while a comprehensive correction force constant, C, represents the combined effects of wall-induced dissipation, particle-network rearrangement, residual elastic recovery, and model simplification. Compared with purely empirical correlations, the proposed model retains a mechanical connection between confined flow and post-exit expansion; compared with comprehensive viscoelastic theories and free-surface simulations, it requires fewer difficult-to-measure parameters and provides a closed-form expression. After calibration under one reference condition, the same value of C is used to predict the swell ratios for different nozzle diameters and nozzle lengths without further adjustment. The model is intended as a physically interpretable and computationally efficient tool for preliminary nozzle selection and free-filament size estimation, primarily in the pronounced-swell regime. Weak-swell and nearly non-swell conditions are outside the reliable predictive range of the current single-parameter formulation.

## 2. Model Development

### 2.1. Deformation Description in a Material Coordinate System

Highly loaded silver paste undergoes pressure-driven axial flow inside the nozzle and experiences radial expansion at the nozzle exit after the release of wall confinement. During flow, the paste may simultaneously exhibit shear deformation, yielding behavior, and rearrangement of the particle network. Therefore, the use of a fixed spatial coordinate system alone is insufficient to accurately describe its deformation history. In this study, a material coordinate system, also referred to as a natural convected coordinate system, is introduced to describe the geometric deformation of paste material points during confined flow inside the nozzle and subsequent swelling at the nozzle exit.

Let $\xi^{i}$ denote the material coordinates. The reference position vector of a material point before deformation is denoted by X, the current position vector after deformation by x, and the displacement vector by u. Thus,

(1) x=X+u

Taking the differential of Equation (1) gives

(2) dx=dX+du

Accordingly, the relationship between the basis vectors before and after deformation can be expressed as

(3) $$g_{i}=G_{i}+\frac{\partial u}{\partial \xi^{i}}$$

where $G_{i}$ is the basis vector in the reference configuration, and $g_{i}$ is the basis vector in the current configuration. This relationship indicates that the flow-induced deformation of the paste inside the nozzle can be mapped to the local variation in the basis vectors through the displacement gradient, thereby providing a geometric foundation for the subsequent development of the outlet stress and swelling-ratio model.

For a cylindrical nozzle, cylindrical coordinates are adopted to describe the flow of the paste. The coordinate system is defined as

(4) $$\{\xi^{1},\xi^{2},\xi^{3}\}=\{r,\theta ,z\}\;$$

If the paste flow is dominated by axial motion inside the nozzle, the displacement components can be simplified as

(5) $$u^{1}=0,\;u^{2}=0,\;u^{3}=(t-t_{0})v_{z}$$

where t is the current time, $t_{0}$ is the reference time, and $v_{z}$ is the axial velocity. It can therefore be seen that, during pressure-driven flow inside the nozzle, the dominant deformation of the paste arises from shear deformation induced by the axial velocity gradient.

**Figure 1:**
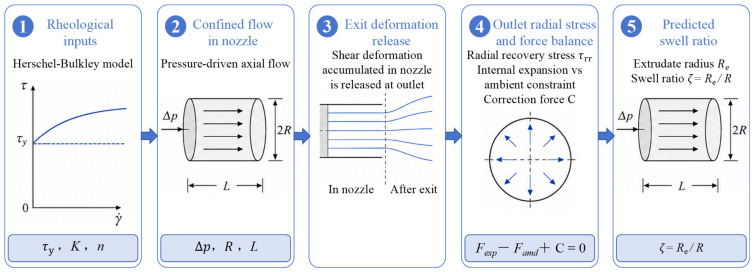
Main flowchart for model development.

Figure 1 illustrates the basic framework for the development of the proposed model. During extrusion, the silver paste undergoes pressure-driven flow and shear deformation inside the nozzle. After the release of wall confinement at the nozzle exit, radial recovery occurs, ultimately leading to an extrusion swelling phenomenon in which the radius of the extruded filament becomes larger than the nozzle radius.

### 2.2. Basic Assumptions and Constitutive Relation

The following simplifying assumptions are adopted:

1. The flow is steady, incompressible, isothermal, axisymmetric, and predominantly axial.
2. A no-slip boundary condition is assumed, and possible wall slip is neglected.
3. Flow in the effective cylindrical nozzle section is approximated as fully developed, while entrance and exit effects are neglected.
4. An unyielded central plug region may exist, but its radius and axial development are not separately resolved.

Because the tested nozzles have different L/D ratios, the fully developed-flow assumption may be less accurate for relatively short or large-diameter nozzles, which constitutes a source of model uncertainty.

These assumptions neglect wall slip, time-dependent structural recovery, local entrance and exit effects, and temperature-dependent rheology, whose combined influence is partly represented by the correction force constant C and discussed further in Section 2.5 and Section 5.

Accordingly, the velocity field of the paste can be expressed as

(6) $$\frac{\mathrm{dz}}{\mathrm{dt}}=v_{z}(r),\;v_{r}=v_{\theta }=0$$

In addition, the fully developed axial flow satisfies

(7) $$\frac{\partial v_{z}(r)}{\partial z}=0$$

Highly loaded conductive silver paste exhibits pronounced yield-stress and shear-thinning characteristics. Therefore, its rheological behavior is described using the Herschel–Bulkley constitutive [14] relation:

(8) $$\tau \;=\;\tau_{y}\;+\;K{\dot{\gamma }}^{n}$$

where τ is the shear stress, $\tau_{y}$ is the yield stress, K is the consistency index, n is the flow index, and $\dot{\gamma }$ is the shear rate. This model captures the non-Newtonian behavior of the silver paste, namely that effective flow occurs only when the applied shear stress exceeds the yield str5ess, and that the apparent viscosity decreases with increasing shear rate.

### 2.3. Expression for Radial Stress at the Nozzle Exit

**Figure 2:**
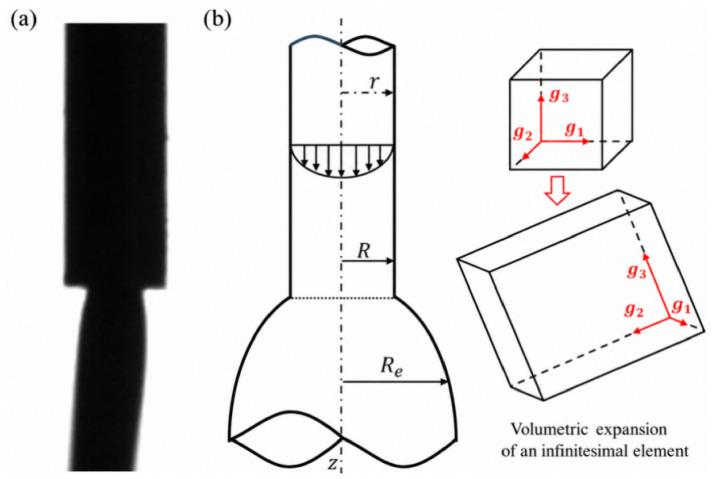
(**a**) Extrusion expansion of non-Newtonian high-viscosity conductive silver paste; (**b**) schematic diagram of extrusion expansion.

The silver paste is driven by the pressure difference Δp inside the nozzle and undergoes shear deformation during axial flow. After the paste exits the nozzle, the originally constrained deformation structure is released, resulting in a tendency toward radial expansion, as schematically shown in Figure 2. The confined flow analysis is based on the Herschel–Bulkley constitutive relation and the established description of pressure-driven flow of yield-stress fluids in cylindrical channels. In addition, the assumption that constrained deformation is partially released after the material leaves the nozzle is consistent with classical die-swell theory and previous numerical studies of Herschel–Bulkley extrudate swell [14,26]. Based on these theoretical concepts, an equivalent radial recovery stress is formulated in the present study. To ensure dimensional consistency, a reference stress is defined as

$$\tau_{ref}=K{\dot{\gamma }}_{ref}^{\;n},\;{\dot{\gamma }}_{ref}=1\;s^{-1},$$

where $\tau_{ref}$ has units of Pa. Based on the above theoretical considerations, the equivalent radial recovery stress is expressed as

(9) $$\tau_{rr}(r)=\tau_{y}+\tau_{ref}{\left[\frac{\frac{3n\Delta p\;r}{2L}-\tau_{y}}{\tau_{ref}}\right]}^{2}$$

where L is the effective nozzle length and rrr is the radial position on the outlet cross-section. The term inside the square brackets is dimensionless, ensuring that $\tau_{rr}$ has units of Pa. Equation (9) indicates that the equivalent radial recovery stress is jointly affected by the rheological properties, feeding pressure, nozzle length, and radial position. A higher feeding pressure promotes radial deformation recovery at the nozzle exit, whereas an increase in nozzle length enhances flow dissipation and weakens extrusion swell.

### 2.4. Modified Model for the Extrusion Swell Ratio

Let R denote the nozzle radius and $R_{e}$ denote the radius of the freely extruded filament after swelling. The radial expansion force induced by the radial recovery stress over the outlet cross-section can be written as

(10) $$F_{r}=\int_{0}^{R}\tau_{\mathrm{rr}}(r)\;2\pi r\;\mathrm{dr}$$

After swelling, the extruded filament is constrained by the ambient atmospheric pressure. The corresponding force can be expressed as

(11) $$F_{p}=\int_{0}^{R_{e}}p_{0}\;2\pi r\;\mathrm{dr}$$

where $p_{0}$ is the ambient atmospheric pressure. Considering that wall-induced energy dissipation, particle-structure rearrangement, residual elastic release at the outlet, and errors arising from theoretical simplification may also occur during the actual extrusion process, a comprehensive correction force constant C is introduced into the radial force balance. Thus,

(12) $$\int_{0}^{R}\left\{\tau_{y}+\tau_{ref}{\left[\frac{3n\Delta p\;r}{2L\tau_{ref}}-\frac{\tau_{y}}{\tau_{ref}}\right]}^{2}\right\}2\pi r\;dr+C=\int_{0}^{R_{e}}p_{0}\;2\pi r\;dr$$

The extrusion swell ratio is defined as

(13) $$\zeta =\frac{R_{e}}{R}$$

Substituting Equation (13) into Equation (12) and rearranging the radial integral gives the modified extrusion swell ratio model:

(14) $$\zeta^{2}=\frac{\tau_{y}}{p_{0}}+\frac{2}{p_{0}\tau_{ref}}\left[{\left(\frac{3n\Delta pR}{4L}\right)}^{2}-\frac{\Delta p\;\tau_{y}R}{3L}+\frac{\tau_{y}^{2}}{2}\right]+\frac{C}{\pi p_{0}R^{2}}$$

Equation (14) is the core expression used for the theoretical calculations in this study. This model simultaneously considers the effects of the Herschel–Bulkley rheological parameters, feeding pressure, nozzle radius, and nozzle length on extrusion swell behavior. It should be noted that C is not an integration constant in the mathematical sense, but a comprehensive correction force constant used to represent the combined effects of factors that are not explicitly modeled in the actual extrusion process.

### 2.5. Calibration of the Correction Force Constant

To clarify the nozzle designation, Nozzles 1–6 refer to six stainless-steel dispensing nozzles arranged in ascending order of their measured outlet diameters, namely 302, 342, 384, 547, 627, and 1100 μm, respectively. All six nozzles have an effective length of 10 mm. Nozzles 1–4 were tested at a feeding pressure of 0.30 MPa, whereas Nozzles 5 and 6 were tested at 0.25 MPa.

According to the experimental results, Nozzles 1–4 exhibited pronounced extrusion swelling, whereas Nozzle 5 showed a small but measurable swell ratio of approximately 1.013, corresponding to the transition between pronounced swelling and nearly no swelling. Nozzle 6 exhibited almost no observable swelling, with a swell ratio of approximately 1.000. Nozzle 5 was therefore selected as the single calibration condition because it produced a stable continuous filament, showed relatively small measurement dispersion, and retained a nonzero swelling response. This condition avoids calibrating the correction parameter using either a pronounced-swell case or the zero-swell boundary represented by Nozzle 6.

By substituting the experimentally measured swell ratio and the corresponding feeding pressure, nozzle radius, effective nozzle length, and rheological parameters of Nozzle 5 into Equation (14), the comprehensive correction force constant was obtained as

(15) $$C\;=1.03\;\times \;10^{-2}\;N$$

After this single-point calibration, the same value of C was retained without further adjustment for the predictions under the remaining nozzle-diameter and nozzle-length conditions. Nozzles 1–4 were used to evaluate the predictive capability of the model in the pronounced-swell regime, whereas Nozzle 6 was used to examine the applicability boundary of the model in the nearly non-swell regime. Although this treatment avoids the introduction of multiple condition-dependent fitting parameters, the use of a single correction parameter may have limited applicability when the operating conditions span pronounced-swell, weak-swell, and nearly non-swell regimes.

## 3. Materials and Methods

### 3.1. Experimental Materials and Micro-Extrusion Platform

The experimental material was a highly loaded conductive silver paste customized by a commercial partner. The mass fraction of silver particles was approximately 90%, and the particles were mainly near-spherical particles with diameters of approximately 3–5 μm. To minimize the influence of material history on the rheological and extrusion results, all experiments were performed using the same batch of paste, with identical sealed storage, pretreatment, and resting procedures. This study focuses on the confined flow inside the nozzle and the free-exit stage, and the formulation composition was not treated as a variable.

**Figure 3:**
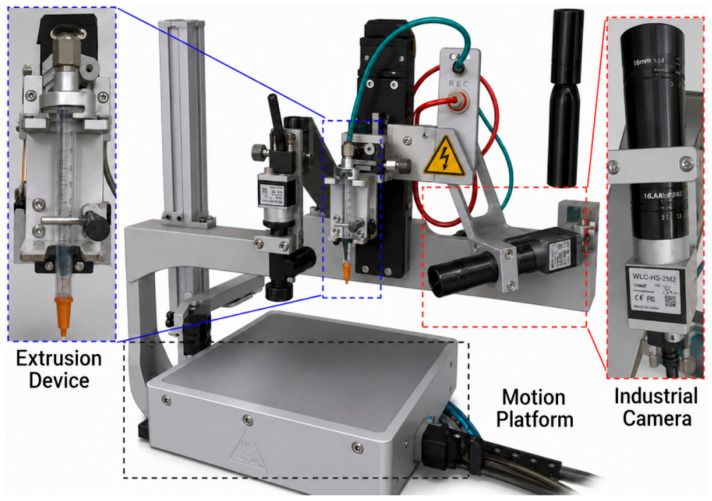
Micro-extrusion experimental platform and image acquisition system: industrial camera, pressure-driven extrusion device, and motion platform.

The experimental platform consisted of a pressure-driven extrusion device, a motion platform, an industrial camera, and an installation and adjustment mechanism, as shown in Figure 3. The pressure-driven extrusion device was used to mount the material barrel and nozzle and to drive the silver paste continuously through the nozzle under stable pneumatic pressure. The motion platform was used to adjust the relative position between the nozzle and the collection region. The industrial camera was positioned near the nozzle outlet to record the formation of the extruded filament and to measure the diameter of the free filament. Before each experiment, the nozzle position, camera focus, imaging direction, and image scale were uniformly adjusted to ensure that both the nozzle outlet and the free-filament measurement region were clearly visible. The free-filament diameter was measured before the filament contacted the substrate, thereby minimizing the effects of platform motion, substrate wetting, and deposition-induced spreading on the measured outlet swelling behavior.

The extrusion pressure was supplied by a domestic precision pneumatic pressure controller (GPC-900, China), with a pressure adjustment range of 0–0.90 MPa and a pressure regulation accuracy of ±0.001 MPa. Before image acquisition, the paste was extruded at the preset pressure for 30 s to ensure the formation of a stable and continuous free filament. Image acquisition was performed using an industrial camera (MER2-503-36U3M, Daheng Imaging, Beijing, China) equipped with a 2× telecentric lens. The image resolution was 2448 × 2048 pixels, and the acquisition frame rate was 30 fps. Before the experiments, the pixel size was calibrated using a standard calibration ruler. During all experiments, the relative positions among the nozzle outlet, free filament, and optical axis of the camera were kept consistent to reduce the influence of imaging angle, focal-length variation, and scale-calibration errors on the free-filament diameter measurements.

### 3.2. Rheological Measurements

Rheological measurements were performed using a rotational rheometer (MCR 302, Anton Paar, Graz, Austria) equipped with a 25 mm parallel-plate geometry. The measurement gap was set to 1 mm, and the test temperature was controlled at 25 °C. The rheological tests included shear stress–shear rate measurements, apparent viscosity–shear rate measurements, viscosity–temperature measurements, and small-amplitude oscillatory frequency sweeps. The corresponding test conditions are listed in Table 1.

**Figure 4:**
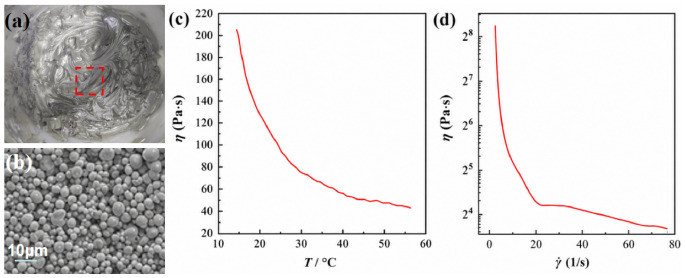
Morphology and steady-state rheological characteristics of conductive silver paste: (**a**) high-solid silver-containing paste(The red box indicates the sampling and characterization region in (**b**); (**b**) nearly spherical micron-sized silver particles; (**c**) viscosity versus temperature; (**d**) viscosity versus shear rate.

To reduce the influence of thixotropy and loading history on the test results, the sample was allowed to rest for 5 min after loading. It was then pre-sheared at a shear rate of 10 s^−1^ for 60 s, followed by another resting period of 180 s before formal testing. During the tests, a solvent trap was used and the sample edges were trimmed to minimize solvent evaporation and edge effects. The shear stress and apparent viscosity as functions of shear rate were measured using a steady shear-rate sweep over a shear-rate range of 0.1–75 s^−1^. The viscosity–temperature test was performed at a shear rate of 1 s^−1^ over a temperature range of 15–55 °C. The small-amplitude oscillatory frequency sweep was conducted over an angular frequency range of 0.1–100 rad·s^−1^, with the strain set to 0.05%. Each rheological test was repeated at least three times to ensure reliability. The results are shown in Figure 4. The obtained shear stress–shear rate curves were used to fit the Herschel–Bulkley model parameters, namely $\tau_{y}$, k, and n, which were subsequently used in the theoretical calculation of the extrusion swell model.

**Figure 5:**
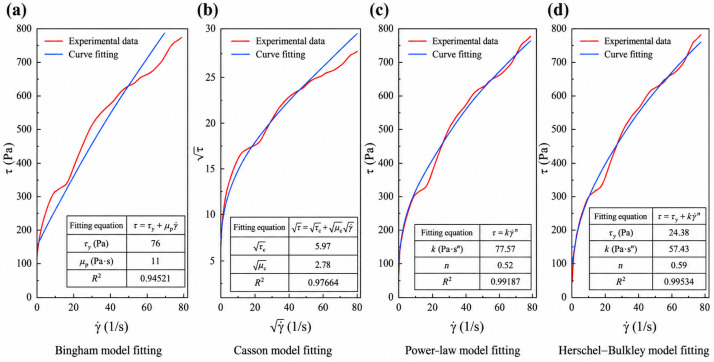
Comparison of different constitutive models in fitting shear stress–shear rate data: (**a**) Bingham model; (**b**) Casson model; (**c**) power-law model; (**d**) Herschel–Bulkley model.

Figure 5 shows the fitting results of different constitutive models for the measured shear stress–shear rate data. The Bingham model provided the lowest goodness of fit ($R^{2}=0.94521$) and failed to capture the pronounced curvature in the low-shear-rate region. The Casson model improved the fitting performance ($R^{2}=0.97664$), but its ability to describe the nonlinear response over the entire shear-rate range remained limited. The power-law model achieved a higher coefficient of determination ($R^{2}=0.99187$); however, it does not explicitly account for the yield-stress behavior of the silver paste. In contrast, the Herschel–Bulkley model incorporates both a yield-stress term and a nonlinear shear-thinning term and exhibits the highest coefficient of determination ($R^{2}=0.99534$) among the four models. The fitted parameters were $\tau_{y}=24.38\;Pa$, $K=57.43\;Pa\cdot s^{n}$, and n=0.59. Therefore, the Herschel–Bulkley model was selected for the subsequent theoretical calculations, in which $\tau_{y}$ was rounded to 24 Pa.

### 3.3. Extrusion Experiments with Different Nozzle Diameters

**Figure 6:**
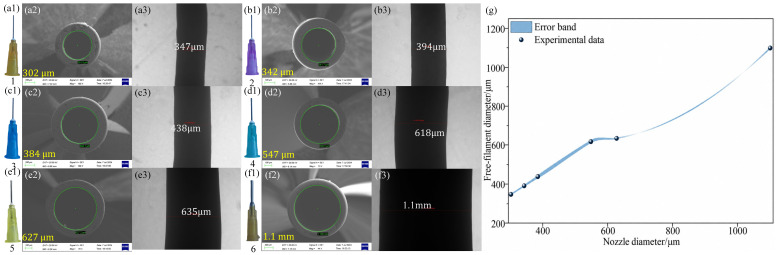
Free-extrusion experiments using nozzles with different outlet diameters: (**a1**–**f1**) physical images of the nozzles; (**a2**–**f2**) scanning electron microscopy images of the nozzle outlets; (**a3**–**f3**) measurements of the free-filament diameter; and (**g**) statistical summary of the experimental results.

To validate the applicability of the model to variations in nozzle diameter, six groups of stainless-steel dispensing nozzles with different outlet diameters were selected for extrusion experiments. Before the experiments, the outlet morphology of each nozzle was observed using scanning electron microscopy, and the actual inner diameter of the nozzle was measured from the outlet images. For each nozzle, at least three clear outlet images were selected, and the outlet diameter was measured in two mutually perpendicular directions. The final nozzle inner diameter was obtained as the average of multiple measurements. For nozzles with slight ellipticity or edge irregularities, an equivalent circular diameter was used as the nozzle diameter in the model calculations to reduce the influence of nozzle manufacturing errors and image-measurement errors on the theoretical results. During the extrusion experiments, the feeding pressure was adjusted using a pressure valve to ensure stable and continuous extrusion for each nozzle. The outlet dimensions, free-filament diameters, and corresponding extrusion swell ratios of the different nozzles are shown in Figure 6 and listed in Table 2.

### 3.4. Extrusion Experiments with Different Nozzle Lengths

**Figure 7:**
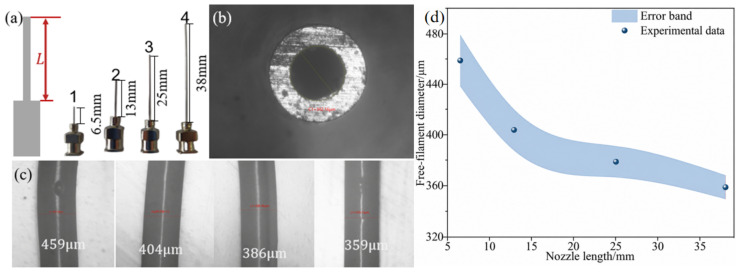
Extrusion experiments with different nozzle lengths: (**a**) Schematic and actual image of the nozzle tube length; (**b**) image of the nozzle outlet; (**c**) measurement results of free-filament diameter; (**d**) statistical plot of experimental results.

To further validate the ability of the model to describe the effect of nozzle length, extrusion experiments were conducted using nozzles with the same outlet inner diameter but different tube lengths. The outlet inner diameter of this group of nozzles was 342 μm, while the nozzle lengths were 6.5, 13, 25, and 38 mm. Because increasing the nozzle length substantially increases the flow resistance, the feeding pressure was adjusted from 0.35 to 0.60 MPa with increasing nozzle length to maintain stable and continuous extrusion. Therefore, this group of experiments was not a strictly single-variable investigation of nozzle length. Instead, it was designed to evaluate whether the proposed model could reproduce the overall variation in extrusion swell under the present coupled nozzle-length and feeding-pressure conditions. Accordingly, the observed changes in the swell ratio should not be attributed solely to nozzle length. The experimental conditions and results are shown in Figure 7 and listed in Table 3.

### 3.5. Data Processing and Evaluation Metrics

The extrusion swell ratio was calculated as the ratio of the free-filament diameter to the nozzle outlet inner diameter:

(16) $$\zeta =\frac{D_{e}}{D}$$

where $D_{e}$ is the experimentally measured free-filament diameter, and D is the nozzle outlet inner diameter. The theoretical prediction error was evaluated using the absolute error, relative error, and mean absolute percentage error (MAPE):

(17) $$E_{a}=\zeta_{theory}\;-\;\zeta_{\exp}$$

(18) $$E_{r}=\frac{\zeta_{theory}\;-\;\zeta_{\exp}}{\zeta_{\exp}}\;\times \;100\%$$

(19) $$\mathrm{MAPE}=\frac{1}{N}\sum_{i=1}^{N}\left|\frac{\zeta_{\mathrm{theory},i}\;-\;\zeta_{\exp,i}}{\zeta_{\exp,i}}\right|\times \;100\%$$

## 4. Results

### 4.1. Model Validation Using Nozzles with Different Diameters

**Figure 8:**
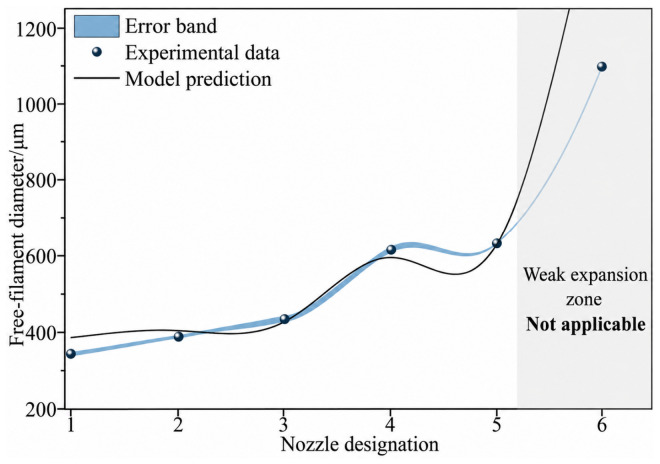
Comparison of the experimental and theoretical extrusion swell ratios for different nozzle diameters. The gray region indicates the weak- or negligible-swell regime, which cannot be reliably described using a single value of C.

Using the correction force constant calibrated from Nozzle 5, $C\;=\;1.03\;\times \;10^{-2}\;N$, the nozzle radius, feeding pressure, and effective nozzle length in the experiments with different nozzle diameters were substituted into Equation (14) for theoretical calculation. The comparison between the theoretical and experimental extrusion swell ratios for different nozzle diameters is shown in Table 4 and Figure 8.

As shown in Table 4, for Nozzles 1–4, which exhibited pronounced swelling, the theoretical swell ratios reasonably captured the experimental trend. For Nozzle 1, the theoretical value was 1.291, which was higher than the experimental value of 1.149. For Nozzle 2, the theoretical value was 1.190, also higher than the experimental value of 1.146. For Nozzles 3 and 4, the theoretical values were 1.122 and 1.092, respectively, slightly lower than the corresponding experimental values of 1.141 and 1.130. Based on the relative errors listed in Table 4, the mean absolute percentage error for Nozzles 1–4 was approximately 5.31%, indicating that the model has a certain predictive capability in the pronounced-swell regime.

When all six nozzles were included in the evaluation, the mean absolute percentage error increased to approximately 11.84%. This increase was mainly caused by Nozzle 6. The outlet diameter of this nozzle was 1100 μm, and its experimental swell ratio was 1.000, indicating nearly no observable swelling. However, the model predicted a swell ratio of 1.495, resulting in a clear overestimation. For Nozzle 5, the theoretical value was nearly identical to the experimental value because this nozzle was used to calibrate C. These results indicate that the current model can reveal the coupled effects of nozzle radius, feeding pressure, and the comprehensive correction force. However, a single value of C is insufficient to simultaneously cover pronounced-swell, transition-swell, and nearly non-swell conditions.

From a physical perspective, the wall shear intensity in a large-diameter nozzle is relatively low, and the accumulation of recoverable deformation in the paste is limited. Meanwhile, the final filament diameter may be more strongly affected by deposition spreading, wetting state, wall slip, outlet-shape stability, and measurement resolution. Therefore, the pronounced overestimation for Nozzle 6 should not be interpreted as a complete failure of the model, but rather as an indication of the applicability boundary of the present single-parameter model. For conditions where the swell ratio approaches unity, further correction terms, such as a zero-swell boundary, slip correction, or relaxation-time term, should be introduced in future work.

### 4.2. Model Validation Using Nozzles with Different Lengths

**Figure 9:**
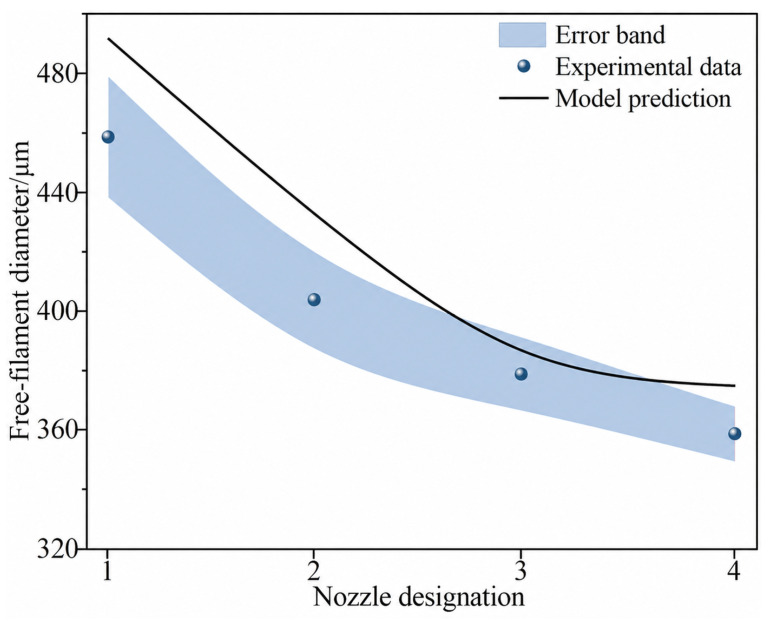
Comparison of the experimental and theoretical extrusion swell ratios for different nozzle lengths.

To evaluate the ability of the model to describe the effect of nozzle length, the experimental conditions listed in Table 3 were substituted into Equation (14) for theoretical calculation. In this group, all nozzles had the same outlet inner diameter of 342 μm, whereas the nozzle lengths were 6.5, 13, 25, and 38 mm. The feeding pressure was increased from 0.35 to 0.60 MPa as the nozzle length increased. The calculation results are shown in Table 5 and Figure 9.

As shown in Table 5, when the nozzle inner diameter was kept constant, the experimental swell ratio decreased from 1.342 to 1.050 as the nozzle length increased from 6.5 to 38 mm. Similarly, the theoretical swell ratio decreased from 1.439 to 1.096. Both the theoretical and experimental results exhibited a decreasing trend with increasing nozzle length, indicating that the modified model can describe the weakening effect of nozzle length on outlet swelling.

Quantitatively, the mean error for the different nozzle-length groups was 0.062, and the mean absolute percentage error was 5.20%. The relative errors of all four groups were within 8%. In particular, for the medium-to-long nozzles with lengths of 25 and 38 mm, the relative errors were 1.99% and 4.38%, respectively, showing good agreement between the theoretical and experimental results. For the two shorter nozzles with lengths of 6.5 and 13 mm, the theoretical values were slightly higher than the experimental values, but the errors remained within an acceptable range.

These results further demonstrate that increasing the nozzle length extends the confined-flow duration of the paste inside the nozzle and enhances wall resistance and flow-induced energy dissipation, thereby reducing the amount of recoverable deformation released at the outlet. Although the feeding pressure was increased with nozzle length in this group of experiments, both the theoretical and experimental swell ratios still decreased, indicating that nozzle length has a clear suppressive effect on extrusion swell within the present experimental range.

## 5. Discussion

### 5.1. Physical Significance of the Model

The novelty of the proposed model lies in the construction of a closed-form mechanical relationship between pressure-driven confined flow inside the nozzle and free radial expansion at the nozzle exit for a highly concentrated yield-stress paste. In Equation (14), the feeding pressure Δp, nozzle radius R, and effective nozzle length L characterize the processing and geometric conditions, while τ_y_, K, and n describe the independently measured yield-stress and shear-thinning behavior of the silver paste. Only the comprehensive correction force C is determined from an extrusion experiment. Therefore, the principal dependencies of the model are physically specified rather than obtained entirely through regression.

Compared with empirical line-width models, the present formulation does not treat the extruded filament diameter merely as a fitted function of nozzle size and processing parameters. Instead, the processing variables enter the model through the pressure-driven flow and outlet force-balance relations. This structure improves the physical interpretability of the predicted effects of nozzle diameter and nozzle length. In particular, the model predicts that an increase in effective nozzle length weakens outlet swelling through enhanced wall interaction and flow-induced dissipation, which is consistent with the experimentally observed decrease in swell ratio from 1.342 to 1.050 as the nozzle length increased from 6.5 to 38 mm.

Compared with classical die-swell models for homogeneous polymer melts, the present framework is adapted to a particulate paste exhibiting yield stress and shear thinning through the Herschel–Bulkley constitutive relation. It does not require direct measurements of the first normal stress difference, relaxation spectrum, or recoverable shear strain, which are difficult to obtain reliably for highly concentrated silver pastes. As shown in the Table 6. Compared with numerical free-surface simulations, the proposed model has a substantially lower parameter and computational burden because the swell ratio can be calculated directly from a closed-form expression after the single calibration of C.

These characteristics make the proposed model particularly suitable for preliminary nozzle-geometry screening, analysis of nozzle-length effects, and estimation of free-filament dimensions within the pronounced-swell regime. After calibration using one reference condition, no additional fitting was performed for the remaining nozzle-diameter and nozzle-length cases. The mean absolute percentage errors were 5.31% for Nozzles 1–4 in the pronounced-swell regime and 5.20% for the nozzle-length group, supporting the ability of the model to capture the primary geometric trends within its applicable range.

Nevertheless, the reduced parameter requirement is achieved by representing several unresolved mechanisms through the comprehensive correction force C. Consequently, the present model cannot resolve local free-surface evolution, time-dependent structural recovery, wall slip, or detailed viscoelastic relaxation in the same manner as a comprehensive numerical model. Its principal advantage is therefore the balance among physical interpretability, parameter economy, and computational simplicity, rather than universal applicability under all extrusion conditions.

### 5.2. Error Sources and Applicability Boundaries

The errors in the current model mainly arise from the following aspects. First, a single constant C is difficult to apply simultaneously to small-diameter nozzles with pronounced swelling, large-diameter nozzles with weak swelling, and nozzles with nearly no observable swelling. In practice, wall shear, particle-network disruption, elastic energy storage, and outlet recovery may vary with nozzle radius and nozzle length. Second, the model simplifies the complex viscoelastic recovery process as an equivalent radial correction force, without explicitly introducing parameters such as the first normal stress difference, relaxation time, thixotropic recovery, and shear history. Third, the experimentally measured free-filament diameter may be affected by local nozzle-outlet morphology and image-measurement resolution. By contrast, the deposited line width may additionally be influenced by substrate wetting, deposition-induced spreading, printing height, and subsequent curing shrinkage. Fourth, the current model does not impose the physical constraint of ζ≥1 and therefore may produce physically unreasonable predictions under certain conditions.

Therefore, the proposed model should be regarded as a semi-theoretical correction model suitable for explaining and preliminarily estimating the swelling trend of highly loaded silver paste in the pronounced-swell regime. It should not be directly claimed to accurately predict the line width under all nozzle-diameter conditions. For large-diameter nozzles with swell ratios close to unity, the corresponding conditions should be treated as weak-swell or nearly non-swell regimes and should be handled using appropriate boundary conditions or piecewise models.

### 5.3. Implications for Micro-Extrusion Processing

From the perspective of process selection, nozzle diameter and nozzle length should not be determined solely according to the target line width. Although small-diameter nozzles are beneficial for improving printing resolution, they are more likely to generate strong wall shear and pronounced outlet swelling, resulting in an actual free-filament diameter larger than expected. Increasing the effective nozzle length can suppress outlet swelling, but it also increases flow resistance and requires a higher feeding pressure. Therefore, in the micro-extrusion of highly loaded silver paste, the target line width, feeding-pressure window, paste rheological properties, and nozzle length should be considered comprehensively. The proposed model can be used for preliminary nozzle-structure screening and free-filament size estimation, but practical process optimization should still be combined with the analysis of post-deposition spreading and curing-induced shrinkage.

## 6. Conclusions

In this study, a single-parameter semi-theoretical correction model was developed to describe extrusion swell during the micro-extrusion of highly loaded conductive silver paste. The main conclusions are as follows:

1. A relationship was established between the extrusion swell ratio ζ, feeding pressure Δp, nozzle radius R, nozzle length L, and rheological parameters based on pressure-driven tube flow and radial force balance. A correction force constant C was introduced to represent the combined effects of wall-induced dissipation, particle rearrangement, residual elastic recovery, and model simplification.

2. The correction force constant was calibrated using Nozzle 5, giving $C=1.03\times 10^{-2}\;N$. For Nozzles 1–4, which exhibited pronounced swelling, the mean absolute percentage error was 5.31%. When all six nozzles were included, the error increased to 11.84%, mainly because of overestimation under the nearly non-swelling condition of Nozzle 6.

3. Under the coupled nozzle-length and feeding-pressure conditions, both the experimental and theoretical swell ratios decreased with increasing nozzle length. The mean absolute percentage error was 5.20%, indicating that the model reproduced the overall decreasing trend under the tested conditions. Because the feeding pressure was varied, this trend cannot be attributed solely to nozzle length.

4. The proposed model should be regarded as a semi-theoretical tool for trend description and preliminary estimation within the tested pronounced-swell range. Its applicability is limited in weak-swell and nearly non-swell regimes, where wall slip, viscoelastic relaxation, shear history, and the physical boundary ζ ≥ 1 may need to be considered.

5. Compared with purely empirical correlations, the model retains a mechanical connection between confined nozzle flow and post-exit expansion while requiring fewer parameters than comprehensive viscoelastic or numerical free-surface models. However, the calibrated parameter and reported accuracy remain specific to the investigated material and processing range.

Future work will examine other concentrated paste and ink systems and incorporate additional effects, such as wall slip, viscoelasticity, thixotropy, curing, and solvent evaporation, when necessary.

## Figures and Tables

**Table 1:** Rheometer test conditions.

Test Item	Measurement Gap	Test Range	Temperature/Shear Condition
Shear stress and viscosity as functions of shear rate	1 mm	Shear rate: 0.1–75 s^−1^	25 °C
Viscosity as a function of temperature	1 mm	Temperature: 15–55 °C	Shear rate: 1 s^−1^
Small-amplitude oscillatory frequency sweep	1 mm	Angular frequency: 0.1–100 rad·s^−1^	Strain: approximately 0.05%

**Table 2:** Statistical results of extrusion at different nozzle diameters.

Nozzle No.	Nozzle Outlet Diameter/μm	Nozzle Length/mm	Feeding Pressure/MPa	Free-Filament Diameter/μm	Experimental Swell Ratio
1	302	10	0.3	347 ± 6	1.149
2	342	10	0.3	392 ± 5	1.146
3	384	10	0.3	438 ± 9	1.141
4	547	10	0.3	618 ± 8	1.130
5	627	10	0.25	635 ± 3	1.013
6	1100	10	0.25	1100 ± 4	1.000

**Table 3:** Experimental parameters for different nozzle lengths.

No.	Nozzle Inner Diameter/μm	Nozzle Length L/mm	Feeding Pressure/MPa	Free-Filament Diameter/μm	Experimental Swell Ratio
1	342	6.5	0.35	459 ± 20	1.342
2	342	13.0	0.50	404 ± 16	1.181
3	342	25.0	0.55	379 ± 12	1.108
4	342	38.0	0.60	359 ± 9	1.050

**Table 4:** Comparison between theoretical calculations and experimental results for nozzles with different diameters.

No.	D/μm	Pressure/MPa	Experimental ζ	Theoretical ζ	Error	Relative Error/%
1	302	0.30	1.149	1.291	+0.142	+12.36
2	342	0.30	1.146	1.190	+0.044	+3.84
3	384	0.30	1.141	1.122	−0.019	−1.67
4	547	0.30	1.130	1.092	−0.038	−3.36
5	627	0.25	1.013	1.010	−0.003	−0.30
6	1100	0.25	1.000	1.495	+0.495	+49.50

**Table 5:** Comparison of theoretical calculations and experimental results for different nozzle lengths.

No.	D/μm	Pressure/MPa	Experimental ζ	Theoretical ζ	Error	Relative Error/%	No.
1	342	6.5	0.35	1.342	1.439	+0.097	+7.23
2	342	13.0	0.50	1.181	1.266	+0.085	+7.20
3	342	25.0	0.55	1.108	1.130	+0.022	+1.99
4	342	38.0	0.60	1.050	1.096	+0.046	+4.38

**Table 6:** Comparison between existing extrusion-dimension modeling approaches and the proposed model.

Modeling Approach	Main Input or Basis	Main Advantages	Main Limitations	Differences in the Present Model
Empirical line-width correlations [5,6,13]	Nozzle size and fitted processing variables	Simple and convenient within the calibrated range	Strong dependence on material, equipment, and operating window; limited physical interpretation	Introduces pressure-driven flow, rheological parameters, nozzle geometry, and outlet force balance
Classical viscoelastic die-swell theories [22,23]	Elastic recovery, normal-stress difference, or recoverable strain	Strong theoretical basis for homogeneous polymer melts	Requires viscoelastic material functions and is difficult to apply directly to highly filled particulate pastes	Uses Herschel–Bulkley yield–shear-thinning rheology and an equivalent correction force
Numerical free-surface models [24,25]	Governing equations, constitutive equations, and detailed boundary conditions	Provides detailed velocity, stress, and free-surface fields	High computational and parameter requirements	Provides a closed-form prediction with one calibrated parameter
Proposed semi-theoretical model	$(\Delta p),(R),(L),(\tau_{y}),$ (K),(n), and (C)	Physically interpretable, low computational cost, and limited calibration requirement	Restricted applicability in weak- and nearly non-swell regimes	Connects confined flow with outlet radial expansion for highly concentrated silver paste

## Data Availability

The data that support the findings of this study are available within the article.
